# Qualitative and quantitative differences in the subgingival microbiome of the restored and unrestored teeth

**DOI:** 10.1111/jre.12642

**Published:** 2019-02-08

**Authors:** Steven W. H. Rademacher, Egija Zaura, Cornelis J. Kleverlaan, Mark J. Buijs, Wim Crielaard, Bruno G. Loos, Marja L. Laine

**Affiliations:** ^1^ Department of Periodontology Academic Centre for Dentistry Amsterdam (ACTA) University of Amsterdam and VU University Amsterdam The Netherlands; ^2^ Department of Preventive Dentistry Academic Centre for Dentistry Amsterdam (ACTA) University of Amsterdam and VU University Amsterdam The Netherlands; ^3^ Department of Dental Materials Academic Centre for Dentistry Amsterdam (ACTA) University of Amsterdam and VU University Amsterdam The Netherlands

**Keywords:** 16S rRNA sequencing, bacteria, metal‐based, restoration

## Abstract

**Background and Objective:**

Metal‐based dental restorations with a subgingival outline may enhance plaque accumulation and bacterial colonization. This study aimed to investigate whether metal‐based restorations influence the composition of subgingival microbiome.

**Material and Methods:**

Per subject one site with a metal‐based restoration and one contra‐lateral site without a restoration were selected on basis of radiographic bone loss ≤2 mm, restoration outline at sulcus level/subgingivally, pocket depth ≤4 mm, and no root canal treatments. Subgingival samples were collected with sterile paper‐points, and microbial profiles were obtained by 16S rRNA gene amplicon sequencing. Restorations were sampled with an Arkansas‐stone and the metal composition was determined using energy‐dispersive X‐ray spectroscopy.

**Results:**

A total of 22 sites from 11 subjects were included. No significant differences for the clinical parameters were found between the restored and unrestored sites. The average age of the restorations was 14.9 ± 7.1 years. *Firmicutes* was the most prevalent phylum at the restored sites (32% vs 20% of the reads of the unrestored sites, *P* = 0.016), and *Actinobacteria* at the unrestored sites (33% vs 18% of the reads of the restored sites, *P* = 0.01). Overall, sequences clustered into 573 operational taxonomic units (OTUs). Species richness of the restored sites was significantly higher than species richness of the unrestored sites (117 ± 32 and 96 ± 20 OTUs, respectively, *P* = 0.013). No associations between the metal composition and bacterial profiles were found.

**Conclusion:**

This study shows that metal‐based restorations may enhance colonization of *Firmicutes* and the neighboring pocket may harbor more diverse microbial communities.

## 1 INTRODUCTION

The placement of a metal‐based dental restoration with a subgingival outline may enhance plaque accumulation and bacterial colonization because of rough surface of the restorative material^1^ and overhang of the restoration.^2^ Gingival tissue cells respond to the bacteria by an inflammatory reaction, which in susceptible subjects may lead to breakdown of periodontal tissues.^3^ It has also been shown that overhanging restorations play a role in the extent of periodontal breakdown.^2^, ^4^, ^5^ Overhanging restorations result in anatomical changes, which may create a more favorable environment for periodontitis‐associated bacteria.^6^, ^7^ Furthermore, galvanic corrosion due to a lower of pH in the crevice^8^ may result in leakage of metal ions into the gingival crevice and tissues.^9^, ^10^ As a result, this could influence bacterial adhesion, toxicity and induce allergies.^11^ On the other hand, metals such as silver (Ag), copper (Cu), gold (Au), and zinc (Zn) have antimicrobial properties and are used for centuries in dentistry and general health care.^12^, ^13^, ^14^

Gingivitis is the first sign of inflammation of periodontal tissues. About 50%‐100% of the adult population can be characterized as having gingivitis.^15^, ^16^, ^17^ Clinical signs of gingivitis are bleeding on probing (BoP), redness and swelling, and gingivitis occurs as a reaction of the gingival tissues to accumulation of bacteria and their toxic products on teeth, which are non‐shedding surfaces. During the development of gingivitis, a shift in the microbiological composition can be seen; gram‐positive cocci and rods are being replaced by gram‐negative cocci, rods, filaments, fusobacteria, and spirochetes.^18^, ^19^

To date, our understanding of the microbiology of gingivitis and microbiological changes in relation to dental restorations is based on traditional targeted techniques. These techniques study the microbiota in a specific manner, *that is,* searching for specific known microorganism only. In vivo studies, using targeted techniques, have shown that *Streptococcus* and *Actinomyces* are the first colonizers of the tooth enamel surface.^20^ With the introduction of open‐ended techniques, it is possible to analyze thousands of bacterial sequences per sample. This technique provides an opportunity to study the entire composition of bacterial communities and identify potentially novel bacterial species in the subgingival biofilm.^18^, ^21^, ^22^ On the basis of 16S rRNA, Griffen et al^21^ identified several new taxa in subgingival plaque of periodontitis patients and patients with a healthy periodontium. Further, they reported that a distinction could be made between subgingival microbiomes of periodontally healthy and diseased sites.

The metal‐based restorations and their actual metal composition may lead to alterations in the subgingival microbiome. We hypothesized that a subgingivally placed metal‐based restoration may influence the composition of the subgingival microbiome. Therefore, the main aim of the present study was to investigate whether and how the subgingival microbiome differs at the sites with a metal‐based restoration compared to the unrestored sites. Furthermore, association of other local factors such as BoP, extent of overhang, age of the restoration, and metal composition, as well as the systemic factor smoking with the subgingival microbiome was analyzed.

## 2 MATERIAL AND METHODS

### 2.1 Subject selection

The current study was designed as a cross‐sectional split‐mouth cohort study and included subjects who visited the clinics of the Academic Centre for Dentistry Amsterdam (ACTA). On the basis of <2 years old existing radiographs (peri‐apical, bitewings or orthopantomograms), subjects were screened for alveolar bone loss ≤2 mm at the targeted sites. The following variables were extracted from the subjects electronic health record: (a) age and gender, (b) diabetes mellitus, (c) smoking habits, and (d) any known (metal) allergies. A subject was defined as a smoker if a current smoker or stopped smoking <1 year ago and as a non‐smoker if a never smoker or stopped smoking ≥1 year ago. Subjects were excluded if they had taken antibiotics or received periodontal treatment in the last 6 months. The Medical Ethical Committee of the VU Medical Centre (VUMC), Amsterdam, approved the study (11/306). All subjects gave a written informed consent, and the study was carried out in accordance with the Declaration of Helsinki.

### 2.2 Selection of the test and control sites

Non‐molar and flat molar surfaces were selected when (a) one tooth site was restored with a metal‐based material (porcelain‐fused‐to‐metal (PFM) crown or amalgam restoration), (b) the outline of the restoration was located at sulcus level or subgingivally, (c) pocket depth was not >4 mm, and (d) no root canal treatment was present. First, the restored site was selected as a test site (eg, tooth 12 mesio‐buccal site) and then the unrestored site of the contra‐lateral tooth (eg, tooth 22 mesio‐buccal site) was selected as a control site. Incisors were the first choice, and then the teeth were checked to the posterior direction until a tooth with a metal restoration was found. If the same site of the contra‐lateral tooth was also restored, the next tooth was selected if unrestored (control site). No metal‐based restorations in the tooth adjacent to the unrestored site were allowed.

### 2.3 Microbial and metal sample collection

Cotton rolls were placed next to the sample sites, and the sampling area was gently air‐dried. Supragingival plaque was carefully removed with a microbrush (Microbrush international, Grafton, WI, USA). The microbiological sample of subgingival plaque was taken from the test and control sites using two sterile paper‐points per site (PP; Absorbent Points # 5‐4; Henry Schein U.K. Holdings Ltd., Southall, Middlesex, UB2 4AU England). The paper‐points were inserted into the pocket for 10 seconds, placed in an empty Eppendorf tube and stored at −80°C until DNA extraction.

After microbial sampling and clinical measurements, a sample of the metal restoration was taken with a new Arkansas‐stone (Dura‐White FL2, Shofu, Japan) at 1000 rpm without water cooling. The restoration sample spot was polished and cleaned after sampling.

### 2.4 Clinical measurements

The clinical measurements at test and control sites were performed after microbiological sampling except for plaque which was recorded before removal of supragingival plaque. Plaque was scored as follows: 0, no plaque; 1, plaque only detectable with a probe; and 2, plaque visually detectable (modified from,^23^ mPI). BoP was scored as follows: 0, no bleeding after probing; 1, bleeding after probing. Mobility was scored according to the Miller classification.^24^ Furthermore, probing pocket depth (PPD), amount of gingival recession (REC), clinical attachment level (CAL), and suppuration were determined at sampled sites. At the test site, the amount of overhang was determined and categorized as follows: −1 = under‐filled, 0 = no overhang, 1 = overhang only diagnosed with the use of a probe, 2 = overhang clinically visible, and 3 = overhang visible on the radiograph. The distance from the most apical part of the restoration outline until the gingival margin was measured. Also the age of the restoration and the type of restoration were assessed. All clinical examinations were performed by the same trained clinician (SR).

### 2.5 DNA extraction, amplicon preparation, and pyrosequencing

DNA from subgingival plaque was extracted as described previously^25^ and quantified on 16S rDNA content through real‐time PCR.^26^ Samples with negative PCR results were subjected to concentration of the DNA through vacuum centrifugation (Martin Christ Gefriertrocknungsanlagen GmbH, Osterode am Harz, Germany). All clinical samples were adjusted to the end DNA concentration of 20 pg/μL and stored at −20°C until further analysis. Besides the clinical samples, DNA extracts from duplicate sterile paper‐points and extraction blanks were included to control for a potential contamination.^27^

Barcoded amplicon libraries of the small subunit ribosomal RNA (16S rRNA) gene hypervariable region V5‐V7 were generated for each individual sample, pooled and sequenced by means of the Genome Sequencer FLX Titanium system (Roche, Basel, Switzerland) as described previously.^26^ The pyrosequencing data were processed and quality filtered; reads containing ambiguous base calls, >1 error in the forward primer, >2 error in the reverse primer, >1 error in the barcode, >6 nt homopolymer sequence, the average quality score below 30, or a length <200 bp or >1000 bp were removed from the analyses. Sliding window test (50 nt) of quality scores was enabled, and sequences of low quality were truncated at the beginning of the poor quality window.^26^ The cleaned reads were clustered in operational taxonomic units (OTUs) at a minimal sequence similarity of 97%, and the representative sequence of each cluster was assigned a taxonomy as described previously.^26^

### 2.6 Analyses of the metals

A modified method for the determination of the composition of the alloys in dental restorations was used.^28^, ^29^ In brief, the composition of the metal particles on the Arkansas‐stone was determined both qualitatively and quantitatively by Energy‐dispersive X‐ray Spectroscopy (EDS) in combination with a high vacuum Scanning Electron Microscopy (SEM) (XL20, Philips/FEI, Eindhoven, The Netherlands). The metal particles of the Arkansas‐stone were transferred onto a carbon tape. Energy of the electron beam was set at 30 keV with a spot size of 6.0. In a high vacuum environment, electrons were fired upon the metal particles resulting in free electrons dispersing energy. The amount of free energy was analyzed with a backscatter electron detector.

### 2.7 Statistical analysis

Statistical analyses were performed using statistical software SPSS PASW Statistics (version 20.0 for Windows, IBM, New York, NY, USA). Microbiome data were tested for normality with Shapiro‐Wilk test. Non‐normally distributed values were log2 transformed. As values for certain parameters were still not normally distributed, Wilcoxon signed ranks test was used to compare paired samples, and Mann‐Whitney *U* test was used to compare independent samples. To normalize for sequencing depth differences, the OTU‐dataset was randomly subsampled (7270 reads/sample in the dataset with 11 sample pairs; 9240 reads/sample in restored and 2030 reads/sample in unrestored sample datasets, Figure S1). On the normalized datasets, the Shannon Diversity Index, Chao‐1 (estimate for total species richness), one‐way permutational multivariate analysis (PERMANOVA; to allow comparisons of microbial profiles between different groups), and the Bray‐Curtis similarity distances between samples were performed using Paleontological Statistics (PAST version 3.02, http://palaeo-electronica.org/2001_1/past/issue1_01.htm) software. Principal Coordinate Analysis (PCoA) plots using weighted UniFrac distances were made using Quantitative Insights in Microbial Ecology (QIIME, version 1.5.0).^30^ *P*‐values <0.05 were considered statistically significant. No corrections for multiple comparisons were made.

## 3 RESULTS

After extensive screening (N = 558) of the Electronic Records of the dental school, a total of 22 subjects were included to the study. From these subjects, both 22 restored and 22 unrestored sites were sampled. A total of 3 subjects were excluded because of negative PCR yield of both restored and unrestored samples (Figure S1). From the remaining 19 subjects, for 11 subjects both the restored and the unrestored site sample (paired) and for 8 subjects only one of the two samples passed the pyrosequencing quality control (Figure S1). The mean age of the 19 subjects was 52 years (±SD 10.5 years; 9 males and 10 females), 13 were Caucasians, 6 were smokers, 2 had diabetes, and none of the subjects reported allergy to metals.

### 3.1 Characteristics of the restored and unrestored sites

Clinical characteristics of the restored and unrestored sites did not differ significantly (Table 1). Mean mPI of the restored sites was 0.8 ± 0.6 compared to 0.9 ± 0.6 at the unrestored sites, and mean BoP on the restored 0.8 ± 0.7 compared to 0.7 ± 0.6 at the unrestored sites. The mean PPD at the restored sites was 2.8 ± 0.9 mm compared to 2.5 ± 0.9 mm at the unrestored sites. Also CAL and mobility scores were similar between the two types of sites. The average age of the restorations was 14.9 ± 7.1 years (range between 7 and 30 years).

**Table 1:** Clinical parameters of the restored and unrestored sites

Clinical parameters (mean ± SD)	Restored site N = 19	Unrestored site N = 19
Modified plaque index	0.8 ± 0.6	0.9 ± 0.6
Bleeding on probing	0.8 ± 0.7	0.7 ± 0.6
Probing pocket depth (mm)	2.8 ± 0.9	2.5 ± 0.9
Recession (mm)	0.0 ± 0.0	0.3 ± 0.8
Clinical attachment loss (mm)	2.8 ± 0.9	2.8 ± 1.1
Mobility	0.1 ± 0.5	0.1 ± 0.3
Distance restoration outline‐sulcus (mm)	0.9 ± 1.0	‐
Overhang	0.7 ± 0.9	‐
Age restoration (years)	14.9 ± 7.1	‐

Table 2 gives an overview of the metal content of the restorations. From the 19 restorations, 14 were PFM crowns and 5 were amalgam restorations. The majority of the PFM crowns (57%) contained copper, 43% gold and silver, 36% zinc and 29% platinum, palladium and chrome. All of the five amalgam restorations contained copper, silver, and mercury, and 80% contained also tin.

**Table 2:** Metal analysis of the 19 restorations. Numbers and percentages of the restorations containing a specific metal

Metal	PFM crown (N = 14)	Amalgam restoration (N = 5)
Gold	6 (43%)	0 (0%)
Platinum	4 (29%)	0 (0%)
Zinc	5 (36%)	0 (0%)
Copper	8 (57%)	5 (100%)
Silver	6 (43%)	5 (100%)
Palladium	4 (29%)	0 (0%)
Chrome	4 (29%)	0 (0%)
Tin	1 (7%)	4 (80%)
Mercury	0 (0%)	5 (100%)

PFM, porcelain‐fused‐to‐metal.

### 3.2 Sequencing output

Of the overall reads, 83% passed the quality control and the denoising and 79% reads (426 240 reads; average length 360 nucleotides) remained after removing of chimeric sequences. The reads of the 19 subjects were classified into 17 phyla. The phyla *Firmicutes, Actinobacteria,* and *Bacteroidetes,* and genera *Streptococcus, Prevotella, Fusobacterium, Corynebacterium,* and *Actinomyces* were the most prevalent microbial taxa in the subgingival plaque.

#### 3.2.1 Sequencing results of unrestored vs restored paired samples

**Figure 1:**
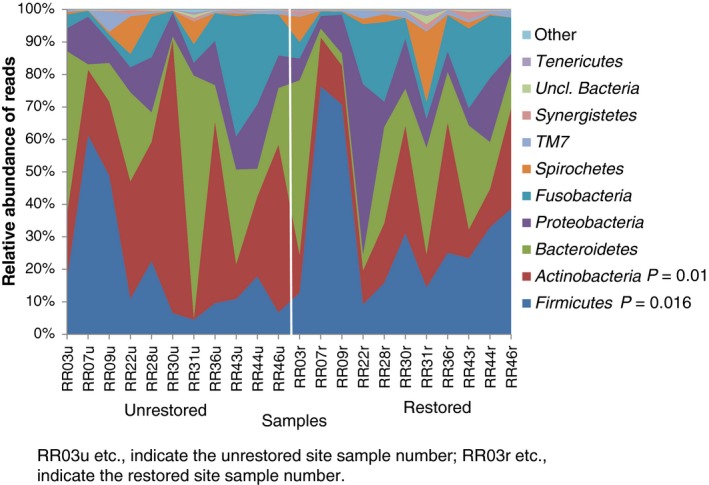
Relative abundance of the major bacterial phyla of the unrestored and restored sites (11 subjects with paired samples). The vertical line separates the individual unrestored sites on the left from the individuals restored sites on the right. The phylum *Actinobacteria* was significantly more abundant at the unrestored sites (*P* = 0.01) while the phylum *Firmicutes* was significantly higher at the restored sites (*P* = 0.016)

First, we addressed the primary aim of the study and compared the microbiomes of paired subgingival samples from 11 individuals with unrestored and restored sites. *Actinobacteria* was the most prevalent phylum at the unrestored sites (33% vs 18% of the reads at the restored sites, *P *=* *0.01). *Firmicutes* was the most prevalent phylum at the restored sites (32% vs 20% of the reads at the unrestored sites, *P *=* *0.016) (Figure 1).

**Figure 2:**
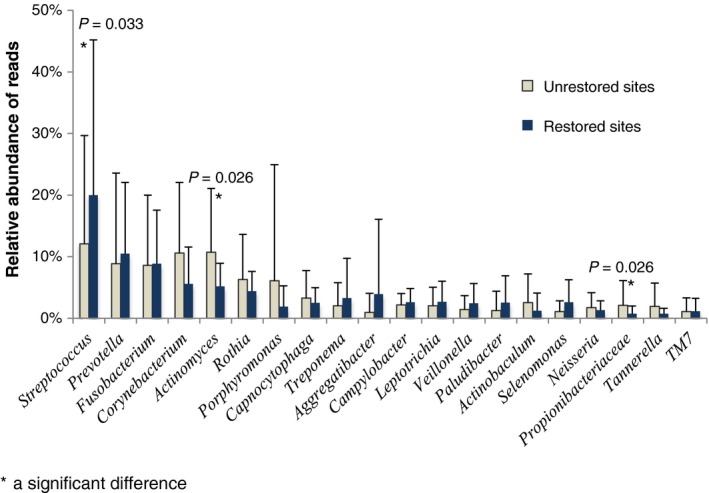
A bar chart of the mean relative abundance for the major bacterial genera (11 subjects with paired samples). The genus *Streptococcus* was significantly increased at restored sites (*P* = 0.033) while the genus *Actinomyces* and family *Propionibacteriacea* were significantly increased at unrestored sites (*P* = 0.026 for both genera). Error bars indicate standard deviation

At the genus level (Figure 2), *Streptococcus* was at a significantly higher proportion present at the restored sites (20% of the reads) compared to the unrestored sites (12% of the reads, *P *=* *0.033). At the unrestored sites, the proportion of genus *Actinomyces* (10% of the reads) and family *Propionibacteriaceae* (2% of the reads) was significantly higher than at the restored sites of the same individuals (5% and 0.5% at the restored sites, respectively; *P *=* *0.026 for both genera).

In total, the sequences could be clustered into 573 OTUs. Significant difference was observed for the total number of OTUs (observed species richness) between the restored and unrestored sites: the restored sites harbored a mean number of 117 ± 32 OTUs in comparison to the 96 ± 20 OTUs at the unrestored sites (*P *=* *0.013). Also the Chao‐1 index that estimates the overall species richness was significantly higher at the restored sites (144 ± 41) than at the unrestored sites (115 ± 32) (*P* = 0.008). However, differences in the Shannon Diversity index did not reach statistical significance (*P* = 0.458) with 2.9 ± 0.81 at the restored and 2.7 ± 0.49 at the unrestored sites. OTUs of *Fusobacterium, Actinomyces,* and *Campylobacter* were detected in all samples.

To reduce the dimensionality of the multivariate OTU‐dataset, PCoA on weighted Unifrac distances was performed. The first (PC1), the second (PC2), and the third (PC3) principal coordinates together accounted for 70% of the variation among the samples (Figure S2). No significant differences were found between the subgingival microbiomes of the restored and unrestored sites (*P* = 0.907, *F* = 0.598; PERMANOVA). This analysis was also performed in relation to the type and age of restoration, different metals present in the restorations, the amount of overhang and smoking (results not shown). Again these analyses did not show any significant differences between the restored and unrestored sites. Interestingly, the microbial profiles of the paired samples from the same individual were more similar than the samples from unrelated individuals at the same type of sites (restored or unrestored): the Bray‐Curtis similarity was significantly higher between the paired samples (0.608 ± 0.06) than the unrelated samples (0.449 ± 0.1) (*P* < 0.001, Mann‐Whitney test).

#### 3.2.2 Sequencing results of all restored samples

In an additional analyses, microbial samples from all restored sites (N = 15, including 11 samples from the subjects with paired samples and 4 samples from the subjects with only a sample from a restored site, Figure S3) were analyzed.

**Figure 3:**
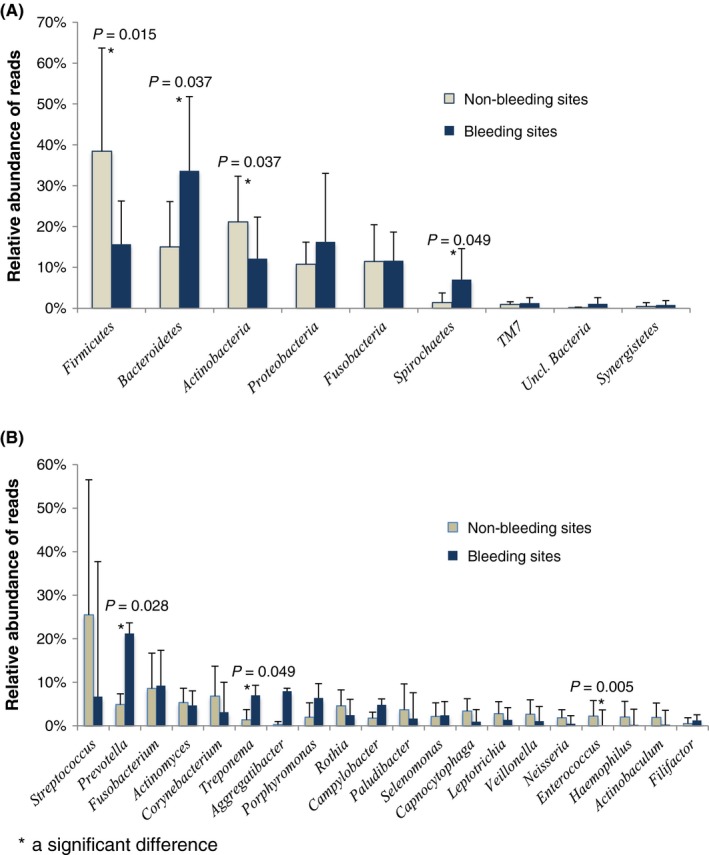
Relative abundance of the major bacterial phyla (A) and genera (B) for the non‐bleeding (N = 7) and bleeding (N = 8) *restored* sites of the 15 subgingival samples. A, The phyla *Firmicutes* (*P = *0.015*)* and *Actinobacteria* (*P *=* *0.037) were significantly increased at the non‐bleeding sites and whereas the phyla of *Bacteroidetes* (*P *=* *0.037) and *Spirochetes* (*P *=* *0.049) were significantly increased at the bleeding sites. B, The genus *Enterococcus* was significantly increased at the non‐bleeding sites (*P* = 0.005) whereas the genera *Prevotella* (*P *=* *0.028) and *Treponema* (*P *=* *0.049) were significantly increased at the bleeding sites. Error bars indicate standard deviation

At the restored sites, there was some spatial discrimination in PCoA between the microbial profiles with and without bleeding (Figure S4), although this difference did not reach statistical significance (*P* = 0.078, *F* = 1.52; PERMANOVA). No significant differences in microbial diversity between the bleeding and non‐bleeding restored sites were observed. However, presence of gingival bleeding (BoP 0 vs 1) was associated with the composition of subgingival microbiome at phylum and genus level. Significantly higher proportion of phylum *Bacteroidetes* and *Spirochetes* was found at the bleeding sites (*P *=* *0.037 and 0.049, respectively; Figure 3A), and *Firmicutes* and *Actinobacteria* at the non‐bleeding sites (*P *=* *0.015 and 0.037, respectively). Further, at the genus level, a significantly higher proportion of *Prevotella* and *Treponema* was found at the restored bleeding sites (*P *=* *0.028 and *P *=* *0.049, respectively; Figure 3B) and *Enterococcus* at the restored non‐bleeding sites (*P *=* *0.005).

#### 3.2.3 Sequencing results of all unrestored samples

An additional analyses were also done for all microbial samples from the unrestored sites (N = 15, including 11 samples from the subjects with paired samples and 4 samples from the subjects with only sample from an unrestored site, Figure S3). No significant differences between the bleeding and non‐bleeding unrestored sites were found in subgingival microbiome profiles, diversity or at the individual taxa level.

## 4 DISCUSSION

The present study is the first study investigating the effect of metal‐based restorations on the subgingival microbiome by an open‐ended technique. The results of this study show that the composition of the subgingival microbiome differs between restored and unrestored periodontal sites, and between bleeding and non‐bleeding restored sites. Further, the subgingival plaque adjacent to metal‐based restorations harbored more diverse microbial communities than the unrestored sites.

A shallow subgingival pocket harbors mainly gram‐positive, facultative anaerobic bacterial species (eg, *Streptococcus* and *Actinomyces*), while in periodontitis a shift to more gram‐negative, obligate anaerobic bacteria (eg, *Prevotella, Fusobacterium* and *Porphyromonas*) is observed.^31^, ^32^ In the present study, a typical subgingival microbiome of a shallow periodontal pocket was found.^33^ However, there were differences between the restored and unrestored periodontal sites; the genus *Streptococcus* (phylum *Firmicutes*) was significantly increased at restored sites while the genus *Actinomyces* and family *Propionibacteriaceae* (both phylum *Actinobacteria)* were significantly increased at unrestored sites. Several authors have reported a relation between surface roughness and bacterial adhesion.^34^, ^35^ However, there is no evidence for preferential adhesion of *Streptococcus* to metals or *Actinomyces* on tooth surfaces. Metal‐based restorations may influence the subgingival microflora by enhanced retention (overhang),^2^ altered adhesion on restoration surface (roughness),^1^ galvanic corrosion,^8^ and leakage of metals to the surrounding crevice.^9^, ^10^ Lang et al^2^ have reported that on‐lays with a proximal overhang increased proportions of gram‐negative anaerobic bacteria, black‐pigmented *Bacteroides,* and anaerobe/facultative ratio. After placement of restorations with clinically perfect margins, a typical microflora for gingival health or initial gingivitis was observed.^2^ The study of Paolantonio et al^6^ showed also a significant reduction of the total bacterial count and the percentages of gram‐negative and anaerobic organisms spreading from overhanging fillings to non‐overhanging fillings.

Metal‐based dental restorations consist of metals like gold, silver, zinc, tin, copper, platinum, mercury, palladium, cobalt, nickel, and chromium.^36^ These metals can be released and found in the oral tissues^9^ and may influence the host immune response.^37^ Next to affecting the immune system, several metals including mercury,^38^ copper and nickel‐chromium,^39^ titanium^40^ and gallium^41^ may induce direct toxic effects. Restoration metals may also have antibacterial activity and consequently alter the composition of the microflora.^13^, ^14^ The current study, however, failed to show any relation between different metals and microbial composition. A larger study sample with similar metal compositions might show influence of metals on the microbial composition.

Bleeding on probing is the first sign of local gingival inflammation. Therefore, we further analyzed our data according to bleeding on probing. At the unrestored sites, no differences in the subgingival microbiome were found between the bleeding and non‐bleeding sites. However, at the restored sites significant differences were found in subgingival microbiomes of the bleeding and non‐bleeding sites. At the bleeding restored sites, higher proportions of the phyla *Bacteroidetes* and *Spirochetes,* and the genera *Prevotella* and *Treponema* were found. Further, at the non‐bleeding restored sites the phylum *Firmicutes*, and the genus *Enterococcus* were found in higher proportions. Since the genera *Prevotella* and *Treponema* are associated with periodontitis,^21^, ^42^ the restored (mean age of the restorations 15 years) sites harboring these microorganisms might be in case of a sudden change in the normal host response more vulnerable for future periodontal breakdown. This could be the result of outgrowth of these bacterial genera or due to the absence or low numbers of health associated bacteria, *for example, Streptococcus* or *Acinetobacter*.^21^

The oral cavity has a large microbial diversity. A previous study on bacterial diversity of 10 periodontally healthy individuals reported on 128 OTUs in a pooled subgingival plaque sample.^43^ In our study, on average 117 and 96 OTUs were found in the subgingival plaque at the restored and unrestored sites, respectively, and the restored sites showed significantly increased number of species richness in comparison to the unrestored sites. Several studies have shown that periodontitis‐associated bacterial communities have increased species richness (=higher taxonomic diversity).^22^, ^44^ The higher bacterial species richness of the restored sites may indicate that these sites are at a higher risk for further periodontal breakdown and require regular periodontal follow‐up. Nevertheless, a large variability was found in the subgingival microbial community structures between and within the subjects. To further explore subgingival microbiomes in various conditions, the future studies should include a larger number of subjects.

In conclusion, the present study showed that metal‐based restorations are associated with enhanced colonization of the bacterial phylum *Firmicutes,* and the bleeding restored sites with enhanced colonization of the phyla *Bacteroidetes* and *Spirochetes*. Further, the neighboring pocket of the restored teeth may harbor more diverse microbial communities. We speculate that altered surface structure and roughness, enhanced retention, galvanic corrosion and leakage of metals may influence microbial composition.
